# Parent–Child Communication after Parental Exposure to Potentially Traumatic Events: A Systematic Review

**DOI:** 10.1177/15248380251343187

**Published:** 2025-06-27

**Authors:** Dani de Beijer, Mèlanie Sloover, Karlijn Heesen, Elisa van Ee

**Affiliations:** 1 Behavioural Science Institute, Radboud University, Nijmegen, The Netherlands; 2 Psychotraumacentrum Zuid Nederland, Reinier van Arkel, Den Bosch, The Netherlands

**Keywords:** trauma, intergenerational transmission of trauma, parenting, PTSD, mental health effects

## Abstract

Intergenerational traumatization poses a risk for the well-being of children whose parents have been exposed to potentially traumatic events (PTEs). Previous research has implied that parent–child communication may significantly contribute to the transmission of trauma across generations, but findings remain limited and inconclusive, particularly regarding the mechanisms and factors that could underlie this process. Therefore, the present paper performed a mixed methods systematic literature review to methodically map how PTE-exposed parents communicate with their children—both in general and about parental PTEs—and how such communication may contribute to trauma transmission. Five electronic databases were accessed to conduct keyword-led searches, yielding a final inclusion of 31 peer-reviewed, empirical studies that investigated parent–child communication among PTE-exposed parents and/or their nonexposed children. Parental PTE exposure was found to have a negative impact on general parent–child communication, often due to the presence of parental anger, irritability, and withdrawal. Conversations about parental PTEs showed substantial diversity in their frequency, content and style, with strategies of partial/modulated disclosure appearing most common. How parents approached PTE communication frequently stemmed from a desire to keep their children safe and unburdened by their previous experiences. Finally, both general communication and PTE communication were implied to contribute to trauma transmission, revealing a significant impact of parent–child communication on child functioning, identity, and well-being. Based on these key findings, the authors discuss meaningful implications for future research (i.e., prospective directions, addressing methodological concerns) and formulate suggestions for clinicians and policymakers surrounding the treatment of PTE-exposed parents and their offspring.

## Introduction

Potentially traumatic events (PTEs) occur frequently across populations worldwide. Described as instances of (in)direct exposure to actual or threatened death, serious injury, or sexual violence (American Psychiatric Association, DSM-5 Task Force, 2013), PTEs vary in shape, ranging from intimate partner violence, sexual assault, and robberies to automobile accidents, natural disasters, and life-threatening illnesses. International prevalence estimates illustrate that over 70% of adults experience at least one PTE throughout their lifetime, with up to 30.5% reporting exposure to four or more (Benjet et al., 2016; Kessler et al., 2017). This exposure, in turn, is associated with a wide range of mental health risks, including (symptoms of) depression, anxiety, substance use, and trauma- and stressor-related disorders, particularly post-traumatic stress disorder (PTSD; Overstreet et al., 2017; Schlaudt et al., 2020; Vallières et al., 2021). Given the evident mental health risks, it is important to investigate how PTE exposure impacts mental health.

### Intergenerational Traumatization

Apart from affecting an individual’s personal mental health, the scathing effects of PTE exposure can also influence subsequent generations, threatening their overall health, well-being, and development (Meijer et al., 2023). This process, known as intergenerational traumatization, broadly refers to the transfer of the effects of trauma from one generation to the next (Kellermann, 2001), placing children of PTE-exposed parents at risk of facing various adverse mental health outcomes despite never being directly exposed to trauma themselves. Contemporary views on intergenerational traumatization describe a complex interplay of various pathways and interactions, conceptualizing the transmission of trauma as a product of psychosocial factors (i.e., attachment, social support, family interactions; Leys et al., 2024; Spiel et al., 2023; Tarabay & Golm, 2024) and (neuro)biological and genetic vulnerabilities (Alhassen et al., 2021; Zhou & Ryan, 2023).

In the specific context of parent-to-child trauma transmission, negative changes in parenting responses due to PTE exposure appear to play an especially salient role (Hartzell et al., 2022; van Ee et al., 2016). Previous research has shown that parents can experience a plethora of negative changes in their behavior post-trauma, giving rise to overprotective and strict responses, increased anger and reactivity, and difficulties expressing affection (Christie et al., 2023). In turn, these behaviors can shape a volatile and distressing climate within the family environment and harm the parent–child relationship, resulting in further detrimental outcomes on both parent and child well-being (Cramm et al., 2022; Meijer et al., 2023). In contrast to these negative consequences, there are also certain parenting strengths that can emerge from PTE exposure and potentially act as protective factors against the transmission of trauma (Siqveland et al.,2012). For example, parents may experience growth in their resilience, improving their ability to cope with adverse circumstances and continue to provide competent, qualitatively strong parenting during and after exposure to duress (Gavidia-payne et al., 2015). The potential for both positive and negative outcomes of parental PTE exposure further illustrates that intergenerational traumatization is a risk, but not an inevitability.

### Parent–Child Communication

One specific parenting response that may play an important part in intergenerational traumatization is the communication between parent and child. Upon being exposed to one or multiple PTEs, forthcoming trauma-related symptoms, including alterations in cognition, mood, and reactivity, can negatively affect how an individual communicates with their social networks (Fredman et al., 2017; Ratcliffe, 2022). Indeed, several studies have implicated parent–child communication as a problem area at risk of dysfunction for PTE-exposed parents (Banneyer et al., 2017; Creech et al., 2017). Parents may exhibit maladaptive communication patterns, which can create confusion and instability for children, consequently impairing intra-family relationships and child attachment security (Dalgaard et al., 2016; Field et al., 2013). Although contemporary findings suggest communication as a crucial element of intergenerational traumatization (Békés & Starrs, 2024), the underlying working mechanisms that explain how communication may give rise to trauma transmission still lack clarification.

### Parent–Child Communication

One specific parenting response that may play a role in intergenerational traumatization is the communication between parent and child. Upon being exposed to one or multiple PTEs, forthcoming trauma-related symptoms, including alterations in cognition, mood, and reactivity, can negatively affect how an individual communicates with their social networks (Fredman et al., 2017; Ratcliffe, 2022). Indeed, several studies have implicated parent–child communication as a problem area at risk of dysfunction for PTE-exposed parents (Banneyer et al., 2017; Creech et al., 2017). Parents may exhibit maladaptive communication patterns, which can create confusion and instability for children, consequently impairing intra-family relationships and children’s attachment security (Dalgaard et al., 2016; Field et al., 2013). Although contemporary findings suggest communication as a crucial element of intergenerational traumatization (Békés & Starrs, 2024), the underlying working mechanisms that explain how communication may give rise to trauma transmission still lack clarification.

The impact of communication may also relate to the content being discussed, particularly when the topic of communication relates to the parental PTE(s). In a therapeutic setting, trauma communication is generally regarded as a challenging but ultimately beneficial, if not a necessary element of healing from trauma-related symptomatology (Lewis-O'Connor et al., 2019). However, findings on talking about PTEs within a family context result in mixed findings regarding its impact on well-being. On the one hand, trauma communication may improve post-traumatic outcomes (Hassija & Turchik, 2016) and enable parents to disclose PTEs to their children. This disclosure can in turn promote closer parent–child relationships and help the child better understand the actions, feelings, and thoughts of their parents (Jeyasundaram et al., 2020). On the other hand, a variety of factors, including the style of communication, the child’s age, and the cultural context, can shape trauma communication to be counterproductive or even harmful to both messenger and recipient (Bonumwezi et al., 2024; Kevers et al., 2024). Dalgaard et al. (2015), for example, illustrated that refugee populations might benefit more from modulated disclosure as opposed to “pushing disclosure in a Western way.” Altogether, the mixed findings stress the need for additional research to critically and comprehensively map trauma communication and its role in well-being and intergenerational trauma transmission.

Despite significant growth in research and forthcoming recognition of the complex relationship between parental PTE exposure and intergenerational traumatization, the role of parent–child communication remains poorly integrated into theory and lacks consensus. Existing studies and reviews of PTE communication often focus on child PTEs or PTEs shared between parent and child (Afzal et al., 2023; Kevers et al., 2024; Sloover et al., 2024), creating a valuable but limited outlook on communication specific to parental PTEs. Furthermore, reviews that exclusively target parental PTEs tend to focus on specific populations or events, reducing the generalizability of findings (Banneyer et al., 2017; Cramm et al., 2022; Creech et al., 2017; Dalgaard et al., 2015). To reach a comprehensive understanding of the relationships between parental PTE exposure, parent–child communication, and trauma transmission, a thorough review of the literature is needed.

### Present Study

The present study aims to systematically review literature on parent–child communication between PTE-exposed parents and their offspring. Parent–child (PTE) communication is a complex process that may pose a serious threat to a child’s well-being due to the risk of intergenerational traumatization. Reviewing current studies on parent–child communication could improve our theoretical understanding of protective and risk factors that influence whether patterns of parent–child communication will contribute to the transmission of trauma. This knowledge may, in turn, be used to inform clinical guidelines dedicated to family functioning or well-being among PTE-exposed parents and their offspring. Altogether, this review addresses three questions: (a) How does parental PTE exposure affect general parent–child communication? (b) How do PTE-exposed parents talk about their PTEs with their children? (c) How does parent–child communication, including communication about parental PTEs, contribute to intergenerational trauma transmission?

## Method

The review was conducted in accordance with the Preferred Reporting Items for Systematic Reviews and Meta-Analyses guidelines (PRISMA; Page et al., 2021). A preregistration of the study was submitted on PROSPERO (ID: CRD42024558379, 13/07/2024).

### Operationalization

As addressed by a previous review on communication about potentially traumatic events (Sloover et al., 2024), the use of relevant terminology is not always consistent across studies on psychotrauma. For the sake of the current review, we therefore adhere to the following terms: First, the term potentially traumatic event (PTE) will be used to refer to any event of actual or threatened death, serious injury, or sexual violence that could potentially result in negative consequences for mental health and well-being. Second, the term general parent–child communication will refer to any measure of communication that assesses the quality, quantity, style, and/or content of communication in a general sense or in relation to observations of (non-PTE related) day-to-day conversations. Subsequently, parent–child PTE communication will refer to any measure of communication specific to the disclosure and discussion of a PTE and/or its consequences on (mental) health. Finally, intergenerational trauma transmission will be used to broadly refer to any measure of parental PTE impact on the child. This impact may relate to child health and (mental) well-being, but also daily functioning, identity, and any other variables used to infer trauma transmission.

### Selection Criteria

In order to be eligible for inclusion, studies had to (a) assess parent–child communication, (b) involve parents with PTE exposure and/or their children, (c) comprise of empirical, peer-reviewed research, and (d) be written in English. Studies were excluded on the grounds of (a) exclusively addressing parent–child communication about child PTEs, (b) only providing assessments of nonverbal communication, (c) focusing on instances of shared trauma, where the topic of interest regarded a PTE shared between parent and child with no prior mention of previous parental PTE exposure, and/or (d) being a single case study, review, conference paper, or dissertation. Rather than limiting eligible research designs, the present review included qualitative, quantitative, and mixed methods studies in order to achieve a holistic overview of the topics of interest.

### Search

#### Search Strategy

Development of the search strategy began with a handsearch on PsycINFO and Google Scholar to obtain five papers with similar or related topics of interest. Upon obtaining a suitable selection, candidate search terms were identified by screening the titles, abstracts, and keywords of all retrieved records. The constructed keywords were then combined into a draft search strategy and fine-tuned through discussion between authors. The resulting string can be found in Supplemental Material File 1. Numerous combinations of subject terms and spellings were used to ensure that all relevant articles were included in the searches.

Records of interest were identified through searches in five health and social care databases: MEDline (1946 to current), EMBASE (1974 to current), PUBMED (1964 to current), PsycINFO (1806 to current), and Web of Science (1945 to current). To identify additional studies, the reference lists of publications eligible for inclusion were examined and subjected to supplementary backward and forward citation searches. All searches took place between February 2024 and April 2024.

#### Screening Process

Screening ensued after findings from each respective search were combined and duplicates were removed. The lead author conducted a primary round of screening by assessing the titles and abstracts of all studies. Upon completion, a secondary round followed during which the lead and second author independently reviewed each article in full text and judged their eligibility. Reasons for inclusion or exclusion were recorded for the sake of comparison. Any disagreements between the two authors were resolved through discussion or by consulting the remaining authors.

### Data Collection and Analysis

Data extraction was conducted by the lead and second author, who worked together to collect and extract data of interest. The following data were extracted: title, bibliographic data (authors, year of publication, journal, country of origin), sample size, demographic characteristics (i.e., age, gender, country of origin, population), research question and specific aims, research methods/study type, measures, type of communication, topics of communication, type of PTE, and study outcomes.

Taking into account the diversity of study designs included in the current review, data analysis adopted a convergent approach whereby qualitative and quantitative findings were simultaneously synthesized to answer the research questions. For research questions 1 and 3, a convergent segregated approach was used, resulting in independent synthesis of quantitative and qualitative data, which were then integrated. Research question 2 instead employed a convergent integrated approach, involving data transformation to combine qualitative and quantitative data. Approaches were chosen based on the nature of the research questions and whether different forms of data addressed the same or different aspects of the relevant topics.

### Risk of Bias Assessment

The Mixed Methods Appraisal Tool (MMAT) by Hong et al. (2018) was employed to assess the quality and risk of bias for each included record. Designed to critically appraise the quality of quantitative, qualitative, and mixed methods studies, the MMAT is deemed a suitable tool for systematic mixed studies reviews. Assessment was conducted by the lead and second authors, who worked independently to rate the eligible studies. Any disagreement encountered was resolved through discussion and reevaluation.

## Results

### Search Results

**Figure 1.**
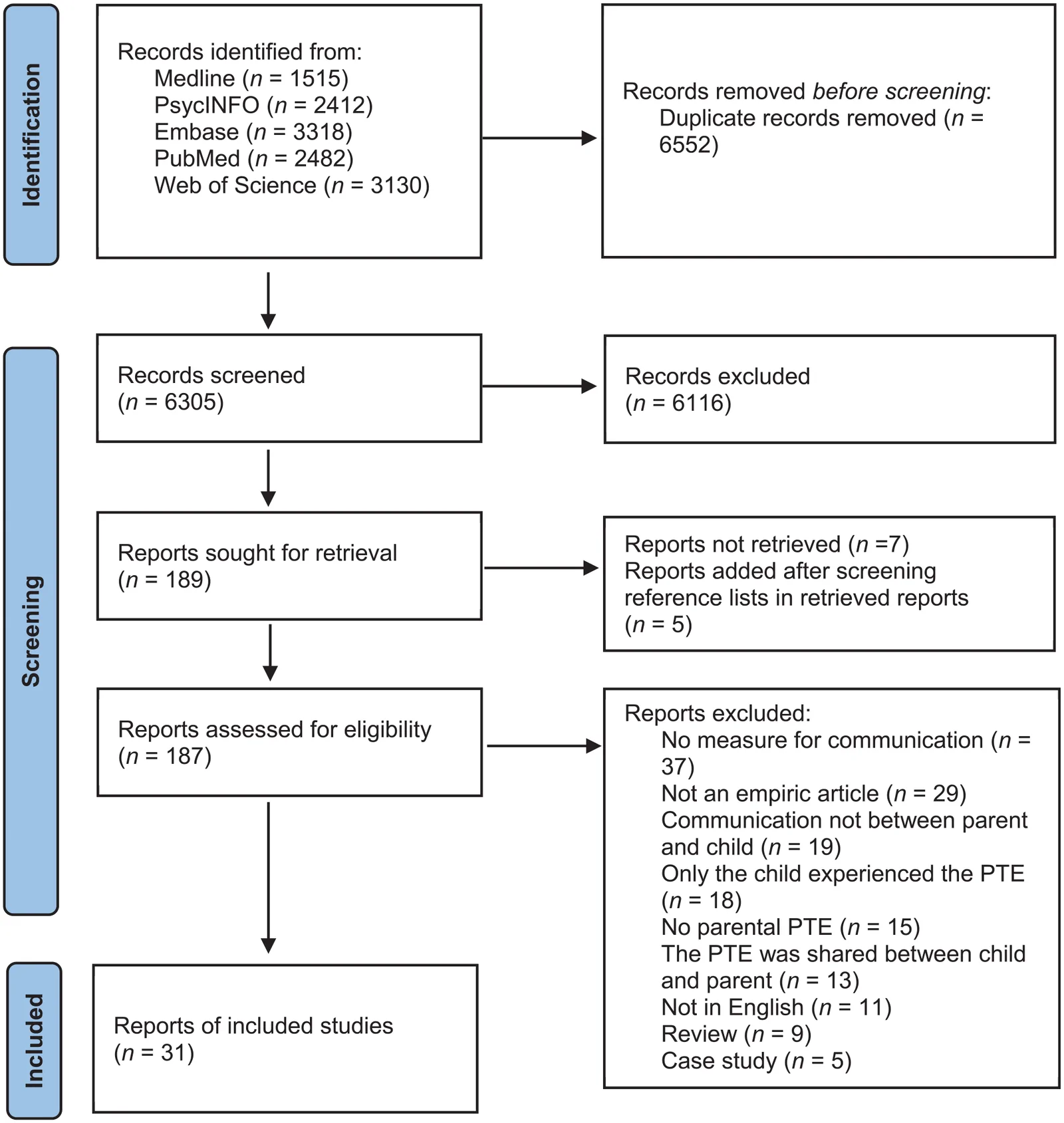
Flow diagram (Page et al., 2021).

The search identified a total of 12,857 records, of which 6,305 remained after the removal of duplicates. After preliminary screening, 189 unique references were sought for retrieval to be screened in full text, yielding a final selection of 31 records. Among the included articles, a majority represented qualitative study designs (*n* = 14), with the remaining studies utilizing quantitative (*n* = 12) or mixed methods (*n* = 5) approaches. A comprehensive summary of the search strategy outcomes can be found in Figure 1.

#### Measurement Assessment

With respect to measurement, a majority of studies adopted (semi-structured) interviews to assess parent–child (PTE) communication, occasionally in combination with focus groups. Interviews and focus groups are considered reliable, valid means to assess lived experiences, and are thus viewed as a suitable method to study parental and child views on communication. Studies with a quantitative component consulted surveys and questionnaires designed to measure parent–child communication and/or parent–child PTE communication. Quantitative measures of general parent–child communication were generally evaluated to be psychometrically sound, while most measures of parent–child PTE communication appeared to lack psychometric evaluation and were occasionally adapted/altered without additional psychometric assessment. These shortcomings limit the overall conceptual and methodological consensus of PTE communication. In these studies, the PTE communication measures did, however, possess a good internal consistency. A more detailed assessment of measurement can be found in Supplemental Material File 3.

### Participant Characteristics

See Table 1 for a full overview of characteristics. The current review encompassed a total sample of 2,644 parents (*M*_age_ = 41.67, SD = 7.42, 56% female) and 3,715 children (*M*_age_ = 22.02, SD = 4.71, 57% female). Records varied greatly in participant country of origin and the type of PTE studied. A majority of PTEs pertained to exposure to war and/or serious conflict (*n* = 23), most commonly the Holocaust (*n* = 10), military deployment (*n* = 6), and the Rwandan Genocide (*n* = 3), followed by the Cambodian Genocide (*n* = 1), the Gaza War of 2008/2009 (*n* = 1), and the Troubles in Northern Ireland (*n* = 1). Other PTEs reported were life-threatening illness (*n* = 2), abduction (*n* = 1), political detainment (*n* = 1), being a refugee (*n* = 1), sexual violence (*n* = 1), the Chernobyl disaster (*n* = 1), and various PTEs (*n* = 2). Data of interest were provided by parents (*n* = 16), children (*n* = 9) or both (*n* = 6).

**Table 1.** Results: The Included Studies.

Article	Type of Study	Country	Type of PTE	Measure of Communication	Participant	*N*	Age *M* (SD)	% Female
Alkalay et al. (2020)	Quantitative	Israel	Holocaust	Comm. of Holocaust experiences (child)	Child	42	46.3 (4.3)	100%
					Parent	–	–	–
Blankenship et al. (2024)	Qualitative	USA	Military deployment	Interviews (parent)	Child	30	< 6	50.2%
					Parent	15	–	52.3%
Bonumwezi et al. (2024)	Quantitative	Rwanda	Genocide	Comm. of Holocaust experiences, revised (child)	Child	251	23.3 (2.4)	–
					Parent	357	–	100%
Bromet et al. (2000)	Quantitative	Ukraine	Chernobyl	CRPBI (parent and child)	Child	300	–	–
					Parent	300	37.2 (4.4)	100%
Christie et al. (2023)	Qualitative	UK	Various	Interviews (parent)	Child	60	23.0 (– )	–
					Parent	30	52.4 (9.1)	53.0%
Dalgaard et al. (2016)	Mixed methods	Denmark	Refugee	Interviews (parent)	Child	30	6.8 (1.6)	46.7%
					Parent	56	–	–
Dalgaard et al. (2019)	Mixed methods	Gaza Strip	War	Custom questionnaire (parent)	Child	170	11.2 (0.8)	49.4%
					Parent	340	37.4 (7.4	50.0%
De Nutte et al. (2022)	Qualitative	Uganda	Forced captivity	Interviews (parent)	Child	–	–	–
					Parent	10	26 to 38	60.0%
Fargas-Malet and Dillenburger (2016)	Mixed methods	Northern Ireland	The Troubles	Custom questionnaire (parent)	Child	73	10.0 (–)	56.2%
		Israel			Parent	73	–	79.5%
Lishner Freud and Berant, (2023) ^a^	Quantitative		Holocaust	HVCI (parent and child)	Child	47	54.7 (3.5)	100%
					Parent	47	–	100%
Gazendam-Donofrio et al. (2009)^b^	Quantitative	The Netherlands	Cancer	PACS (parent)	Child	124	11.0 (3.9)	55.0%
					Parent	124	42.8 (–)	52.4%
Houston et al. (2013)	Quantitative	USA	Military Deployment	Custom questionnaire (child)	Child	13	11.0 (–)	31.0%
					Parent	13	–	100%
Huizinga et al. (2005) ^c^	Quantitative	The Netherlands	Cancer	PACS (parent)	Child	212	15.1 (2.3)	57.0%
					Parent	205	–	84.0%
Kagoyire et al. (2023)	Qualitative	Rwanda	Genocide	Interviews (child) & focus groups (parent)	Child	19	–	–
					Parent	36	–	–
Kav-Venaki et al. (1983)	Qualitative	–	Holocaust	Interviews (parent and child)	Child	15	–	46.7%
					Parent	60	–	50.0%
Kellermann (2008)	Quantitative	Israel	Holocaust	Custom Questionnaire (child)	Child	273	–	69.0%
					Parent	–	–	–
Krauskopf et al. (2023)	Qualitative	Australia	Holocaust	Interviews (child)	Child	24	60.8 (7.7)	54.0%
					Parent	19	–	47.0%
Lin and Suyemoto (2016)	Qualitative	Cambodia	Genocide	Interviews (child)	Child	12	18 to 25	46.2%
					Parent	–	–	–
May et al. (2023)	Qualitative	USA	Veteran	Interviews (parent and child)	Child	7	9 to 17	–
					Parent	7	–	–
McGaw and Reupert (2022)	Qualitative	Australia	Veteran	Interviews (child)	Child	8	14.8 (1.7)	50.0%
					Parent	8	–	37.5%
O’Brien et al. (2019)	Qualitative	Ireland	Sexual violence	Interviews (parent)	Child	–	11.4 (8.8)	–
					Parent	11	45 (9.0)	0.0%
Ochoa et al. (2022)	Quantitative	USA	Various	PACS (parent)	Child	456	13.9 (1.4)	52.9%
		USA + Israel			Parent	456	43.2 (6.6)	92.9%
Okner and Flaherty (1989) ^d^	Quantitative		Holocaust	Custom questionnaire (child)	Child	192	34.3 (– )	53.0%
					Parent	–	–	–
Palgi et al. (2015) (Study 1)	Quantitative	Israel	Holocaust	FIQ (child)	Child	36	57.1 (6.5)	65.7%
					Parent	72	–	–
Palgi et al. (2015) (Study 2)	Quantitative	Israel	Holocaust	FIQ (child)	Child	92	54.6 (4.7)	57.6%
					Parent	–	–	–
Prince (1985)	Qualitative	–	Holocaust	Interviews (child)	Child	20	–	–
					Parent	–	–	–
Rosenheck (1986)	Qualitative	USA	Veteran	Interviews (parent and child)	Child	12	32.4 (6.3)	58.0%
					Parent	5	61.0 (–)	0.0%
Saleh et al. (2019)	Qualitative	Israel	Forced captivity	Interviews (child)	Child	31	16.2 (2.5)	58.0%
					Parent	16	–	–
Sherman et al. (2015)	Mixed methods	–	Veteran	Interviews & focus groups (parent)	Child	–	11.1 (6.0)	–
					Parent	19	39.1 (6.9)	11.0%
Sinalo et al. (2021)	Quantitative	Rwanda	Genocide	Custom questionnaire (parent)	Child	1073	20.6 (9.8)	–
					Parent	317	–	50.0%
Wiseman et al. (2002) ^d^	Quantitative	Israel	Holocaust	Comm. of Holocaust experiences (child)	Child	56	39.7 (4.0)	46.4%
					Parent	56	68.9 (–)	100%
Wolf (2019)	Qualitative	–	Holocaust	Interviews (child)	Child	35	–	–
					Parent	–	–	–

*Note.* Descriptives are based on what was reported in the respective papers.

a Descriptives are based on the oldest two generations (not grandchildren).

b Patient and partner descriptives were combined into one parent descriptive.

c Descriptives are solely based on the clinical sample.

d Descriptives are based on the combined samples.

**Table 3.** Summary of Implications for Practice, Policy, and Research.

Implications
Mental health professionals working with PTE-exposed parents could benefit from integrating psychoeducation surrounding parent–child (PTE) communication into treatment. When formulating advice for parents on parent-child (PTE) communication, it is important to take into account contextual factors such as child age, culture of origin, and alternative sources of information. There is a need for additional research on the link between parent–child communication and parental PTE exposure among parents exposed to events that are not war or conflict related (i.e., sexual violence, domestic abuse, vehicular accidents). Researchers focused on parent–child (PTE) communication among PTE-exposed parents should strive toward greater conceptual consensus and methodological rigor.

**Table 2.** Key Findings of the Systematic Review.

Key Findings
Parental exposure to PTEs was overall found to negatively impact both the quality and quantity of general parent–child communication. Patterns of parent–child PTE communication were highly diverse, with the most common strategy being partial/modulated disclosure, although patterns of silencing were also common. Both general parent–child communication and PTE communication were found to contribute to the transmission of trauma from parent to child by negatively affecting child well-being, functioning, and identity.

*Note. “PTE”* *=* *potentially traumatic event.*

### Bias Assessment

MMAT scoring for each study is reported in Supplemental Material File 2. Risk of bias assessment revealed that a majority of included reports adhered to all quality criteria (*n* = 19). Studies that did not yield fully satisfactory outcomes either had inconclusive (“can’t tell”; *n* = 5) and/or negative (“no”; *n* = 8) ratings on at least two criteria. Closer inspection of the risk of bias across studies unraveled several patterns of interest relating to study design. Most notably, four of the five mixed methods studies included did not satisfy their respective criteria, in particular criterion 5 (assessment of whether study components adhere to quality criteria of each tradition of the methods involved). Among the quantitative studies, half received at least two inconclusive and/or negative ratings, commonly due to a risk of nonresponse bias (*n* = 4). Finally, assessment of the qualitative studies resulted in the highest proportional satisfaction rate, with only three studies receiving unsatisfactory scores. These scores often arose from a lack of necessary information reported in the given articles, which may in part be explained by their older date of publication (1983–1986).

### Impact of Parental PTE Exposure on General Parent–Child Communication

The current review identified 15 articles that assessed facets of parent–child communication not directly related to communication about PTEs. The majority of those studies (*n* = 11) showed results that seem to point to a negative impact of parental PTE exposure on parent–child communication. First, four quantitative studies inferred a negative impact of parental PTE exposure on the quality and/or quantity of parent–child communication. These outcomes originated from cross-comparisons between PTE-exposed parents (adverse childhood experiences and holocaust survivors; Ochoa et al., 2022; Palgi et al., 2015) and nonexposed parents, comparisons between PTE-exposed groups (US and Israeli Holocaust survivors; Okner & Flaherty, 1989) and assessments before and after PTE exposure (military deployment; Houston et al., 2013). Confirmatively, all relevant qualitative records mentioned concerns, impairments, and/or challenges surrounding parent–child communication reported by PTE-exposed parents or their offspring during semi-structured interviews. These findings were visible among different PTE exposures, including military deployment (Blankenship et al., 2024; May et al., 2023; Mcgaw et al., 2022), exposure to genocide (Kagoyire et al., 2023), forced captivity (Saleh et al., 2019), sexual violence (O’Brien), and unspecified PTE exposure resulting in PTSD (Christie et al., 2023). It could therefore be reasonably inferred that, according to these studies, parental PTE exposure does negatively impact parent–child communication.

However, three quantitative studies did not find significant differences in communication quality when comparing PTE-exposed parent–child dyads to a nonexposed comparison group (Alkalay et al., 2020; Bromet et al., 2000; Huizinga et al., 2005). Huizinga et al., (2005) who viewed communication in a sample of adolescent children with parents suffering from cancer, found that adolescents communicated less openly with ill parents, but clinical relevance was deemed small. Similar to these studies, Gazendam-Donofrio et al. (2009), who also investigated parent–child communication among parents with cancer, did not find any significant changes in communication during the first year after parents were diagnosed. Families confronted with parental cancer were found to consistently communicate openly and with few problems, similarly to Chernobyl survivors (Bromet et al., 2000). Altogether, these studies illustrate that parental PTE exposure does not always elicit changes in parent–child communication. It should be noted that the two studies dedicated to cancer patients pertained ongoing PTEs, as the parents were still ill at the time of assessment (Gazendam et al., 2009; Huizinga et al., 2005).

#### Factors Impacting General Parent–Child Communication Quality and Quantity

Several factors were found to interfere with the perceived quality and/or quantity of parent–child communication. Most commonly mentioned was the presence (or expectation) of parental verbal anger, irritability, or harshness, which caused both parents and children to become more apprehensive about communicating with each other (Blankenship et al., 2024; Christie et al., 2023; Kagoyire et al., 2023; May et al., 2023). Some parents described intentionally distancing themselves from their offspring as a result of their anger and limiting social interactions in an attempt to shield their children from harm (Christie et al., 2023; Kagoyire et al., 2023; May et al., 2023). Parental withdrawal and distance were deemed to occur commonly in general (Mcgaw et al., 2022; O'Brien et al., 2019). Children described their parents as noncommunicative, withholding, or emotionally closed (Mcgaw et al., 2022), quoting an inability to respond attentively or address asks for help (May et al., 2023). Similarly to their parents, children also described having concerns for the other’s well-being, which motivated them to not share personal worries and negative feelings (May et al., 2023, Mcgaw et al., 2022, Saleh et al., 2019).

In multiple studies, the distinct type of parental PTE seemed to play a role in parent–child communication. Three articles illustrated how circumstantial restraints tied to the PTE created physical barriers (i.e., distance during deployment, parental detention) that restricted the available frequency and means of contact (Blankenship et al., 2024; Houston et al., 2013; Saleh et al., 2019). Striving to maintain frequent communication through available mediums (i.e., technology, visitation hours) was deemed important to upholding qualitatively strong communication in these cases. In two studies, the specific type of PTE was related to marked differences in communication content and/or quality. Christie et al. (2023) found that some parents who were specifically victims of child maltreatment had very frank conversations about the body with their children, although this finding was based on a small sample size. Subsequently, Huizinga et al. (2005) noted that children of cancer patients encountered greater problems in parent–child communication when parents were facing recurrent disease. Finally, differences in communication could also occur when comparing parents with similar PTE exposures. In a study by Okner and Flaherty, (1989) it was found that children of Israeli holocaust survivors (HS) reported more parent–child communication than the children of American HS, suggesting cultural differences could also attenuate or influence the relationship between parental PTE exposure and parent–child communication.

### Parent–Child Communication about Parental PTEs

Twenty-five studies addressed parent–child communication specific to parental PTEs. Conversations about parental PTEs were generally described as difficult, highly emotional, and intense by both parents and children. Whether parents engaged in parent–child PTE communication in the first place varied greatly within and across records, as addressed by a multitude of studies who described encountering noteworthy diversity in the frequency, style, and content of PTE conversations (De Nutte et al., 2022; Krauskopf et al., 2023; Prince, 1985; Wolf, 2019).

#### Frequency and Depth of PTE Communication

Overall, parents were more likely to (partially) disclose their PTEs to their children rather than remaining fully silent (Alkalay et al., 2020; Bromet et al., 2000; De Nutte et al., 2022; Lin & Suyemoto, 2016). Among records that specifically reported on the frequency of parent–child PTE communication, all but one study found that a majority of parents did at least talk about their PTEs to some degree (Dalgaard et al., 2016, 2019; Fargas-Malet & Dillenburger, 2016; Kellermann, 2008; Sinalo et al., 2021; Wiseman et al., 2002). The frequency of PTE communication could also differ between families according to the country of origin and specific type of PTE experienced. For example, Holocaust concentration camp survivors were more likely to talk about their PTEs when compared to ex-partisans and other Holocaust survivors (Kav-Venaki et al., 1983; Lishner Freud & Berant, 2023), and survivors living in Israel spoke more frequently about their PTEs than survivors in the United States (Okner & Flaherty, 1989). These differences were hypothesized to be partly related to the salience of certain events (i.e., concentration camp may have been more impactful than being ex-partisan) and cultural customs regarding the discussion of certain PTEs, such as the Holocaust being more “present” and commonly discussed in Israel. While a majority of parents appeared to communicate (partially) about their PTEs, patterns of nondisclosure/silencing, described as instances where parents shared very little to no information about their PTE to their child, were also commonplace (Kagoyire et al., 2023; May et al., 2023; Sherman et al., 2015; Wolf, 2019). Three studies focusing on parents with PTSD illustrated that a majority did not speak to their children about their PTEs at all (Christie et al., 2023; McGaw et al., 2022; Rosenheck, 1986), signifying that parents with PTSD in particular may be less likely to engage in parent–child PTE communication.

#### Open and Partial Disclosure of Parental PTEs

In 14 studies, more information was provided about how parents engaged in parent–child conversations about their PTEs. Patterns of PTE communication could differ in the amount of information provided to the child and the strategies adopted to approach these conversations. Three studies described the use of “open disclosure”; a style of communication characterized by greater detail about parental PTEs and the addressing of mental health consequences that these events may have brought forth (Dalgaard et al., 2016; Wolf, 2019). Bromet et al. (2000) found that many Chornobyl survivor parents addressed their trauma through open discussion and grief, emphasizing the open expression of emotion as a core facet of this interaction. A more common approach to PTE communication involved “partial” or “modulated disclosure”; a style of communication characterized by parents being selective of the content being shared. Among the eight studies who described this strategy, instances of partial/modulated disclosure would generally take one of two shapes: (1) discussions of PTEs while omitting certain parts of the story or only addressing specific aspects (i.e. disclosing PTSD but not the cause; Christie et al., 2023; Dalgaard et al., 2016, 2019; Sherman et al., 2015; Wolf, 2019), or (2) the construction of alternative narratives/stories that would allow for communication about the PTE while withholding context and avoiding explicit sharing (Kagoyire et al., 2023, May et al., 2023; Prince, 1985; Wolf, 2019;). For example, Wolf (2019) found that some parents who survived the Holocaust would disclose their PTEs through fairytale-like stories of humor and adventure.

When parents choose to speak up about their PTE(s), the content of conversations could also vary. In a sample of parents with Palestinian national trauma, Dalgaard et al. (2019) found that children were most commonly engaged in conversations about the facts and reasons of war (i.e., talking about the reasons for the losses, wars, and deportation of Palestinians) and violence and aggression, in particular the wrongdoings enacted by the enemy. A small number of parents were found to emphasize positive resources and future prospects in their recollections of war and trauma, with their stories including memories of people helping Palestinians and neighborhoods and villages caring for each other. Other studies also reported parental attempts to present positive messages in their PTE communication (De Nutte et al., 2022; Kav-Venaki et al., 1983; Prince, 1985; Sherman et al., 2015; Wolf, 2019). These manifested as positive life lessons and coping strategies learned from prior trauma, reassurances that the PTE is in the past and has been dealt with, turning memories of PTEs into humorous and adventurous stories, and ensuring children that they are not to blame for current circumstances, including parental mental health.

The choice to engage in parent–child PTE communication was made according to various considerations. Many parents acknowledged the importance and potential benefits of talking about their PTEs, expecting it to positively affect their own well-being, their child’s well-being, and the parent–child relationship (De Nutte et al., 2022; Kagoyire et al., 2023; May et al., 2023;; Sherman et al., 2015). Parents shared the hope that talking about their PTEs would provide children with better insight into parental mental health (particularly PTSD; May et al., 2023) or external stressors such as difficult living conditions and the presence of discrimination and stigmatization (De Nutte et al., 2022). How and when PTE communication took place could also be influenced by the developmental appropriateness of talking about PTEs with children. Parents regularly stressed the importance of adequate timing: It was often deemed more suitable to discuss PTEs with teenagers, adolescents, or adult children than with young children (De Nutte et al., 2022; Fargas-Malet & Dillenburger, 2016; Lin & Suyemoto, 2016; May et al., 2023; Prince, 1985; Sherman et al., 2015; Wolf, 2019). Moreover, discussing PTEs with younger children would be done in a different way: Parents were mindful of the content and language they used and also modulated their degree of disclosure depending on the perceived age-appropriateness of certain topics.

#### Indirect and Nondisclosure of Parental PTEs

In 13 studies, more information was provided about how parents maintained silence and/or avoided speaking about their PTEs with their children. These instances were characterized by patterns of complete withholding or minimizing information to the “bare minimum” that had to be told (Dalgaard et al., 2016; Kagoyire et al., 2023; Krauskopf et al., 2023). For some parents, the decision to remain silent was agentic and deliberate, while other parents felt unable to talk about their PTEs (Wolf, 2019). When children raised topics or asked questions related to the PTE, some parents resorted to distraction or feigned not knowing the answer in order to dismiss the conversation (Dalgaard et al., 2019). Similarly, May et al. (2023) found that some parents with PTSD created false narratives to hide their current state from their children. Parents who wish to remain silent may still disclose accidentally or unknowingly. This was evidenced by Dalgaard et al. (2016), who found that several parents who reported not speaking about their traumatic events with their children nevertheless engaged in open discussions about their PTEs in close proximity to their offspring. Lastly, some parents expressed a desire to disclose their PTEs to their children but could not or did not want to do so face-to-face. Instead, they relied on intentionally having conversations about the PTEs with other adults while their child could overhear (Rosenheck, 1986; Prince, 1985).

Trajectories of silencing were motivated by a variety of beliefs and worries that rendered parents apprehensive or unwilling to engage in parent–child communication about PTEs. Most common was the fear that PTE communication would distress the child and/or negatively impact their mental health (Christie et al., 2023; Kagoyire et al., 2023; May et al., 2023; Mcgaw et al., 2022; Rosenheck, 1986; Sherman et al., 2015; Wolf, 2019). In two studies, the belief that disclosure could be harmful was accompanied by the view that silencing and the resulting forgetting of a PTE would give rise to healing and a means of moving on from the endured trauma (De Nutte et al., 2022; Lin & Suyemoto, 2016). Some parents may thus view avoidance of communication about parental PTEs as a helpful strategy to move on from a traumatic past and prevent harming their children. Other factors that motivated silencing included parental shame, the belief that children were too young or otherwise unable to understand, and the fear that discussing parental PTEs would negatively alter the child’s view of the parent (Fargas-Malet & Dillenburger, 2016; May et al., 2023; McGaw et al., 2022; Rosenheck, 1986; Sherman et al., 2015).

Important to note is that the strategies used to engage in PTE communication were not stagnant and could change over time. One salient factor that could inspire change was the parent’s relationship with their potentially traumatic past. On the one hand, perceived healing and/or distance from past PTEs could give rise to disclosure and more sharing among families where PTEs were previously silenced or (partly) avoided in conversation. On the other hand, PTE communication also took place more frequently when parents faced recent or current PTE exposure, had a history of repeated PTE exposure or were in a period of commemoration related to the endured PTE (Dalgaard et al., 2016, 2019; Fargas-Malet & Dillenburger, 2016; Kagoyire et al., 2023). Changes in PTE communication could also be enabled by external factors, such as children growing older, resulting in more frequent disclosing and greater sharing (May et al., 2023; Sherman et al., 2015). Additionally, the presence of outside sources capable of providing information about the PTE played a role (De Nutte et al., 2023; Kagoyire et al., 2023; Lin & Suyemoto, 2016; Sinalo et al., 2021). In these situations, parents preferred to be the first to “speak up” rather than allowing their children to learn about the PTE from other sources. This did cause the disclosure not to feel like a choice of their own.

### Parent–Child Communication and Intergenerational Trauma

#### Trauma Transmission and Parent–Child Communication in General

Of the 31 articles included, only seven studies observed the potential role of general parent–child communication in intergenerational trauma transmission. Four studies adopted a quantitative approach to assess the impact of communication quality (*n* = 3) or quantity (*n* = 1) on child well-being. All studies assessing quality found that children who reported a lower quality of communication faced more mental health difficulties, which included greater PTSD symptomatology (Huizinga et al., 2005), externalizing behaviors (Ochoa et al., 2022), and internalizing problems and inattention/hyperactivity (Houston et al., 2013). A higher quantity of general communication was found to result in both positive and negative outcomes, with offspring of Holocaust survivors reporting less demoralization, depression, and anxiety, but also more guilt (Okner & Flaherty, 1989). Within this sample, the frequency of communication was found to have less impact on Israeli children when compared to those born and raised in the USA. The role of communication in trauma transmission may thus also differ across societies and/or cultures.

Three studies took a qualitative approach to assess whether parents and/or children noticed changes in child well-being in relation to parent–child communication. Assessments took place through semi-structured interviews and focus groups. Similarly to the quantitative results, each study found that children and/or parents believed that the parental trauma had impacted child well-being, and that parent–child communication had played a role in enacting this “ripple effect” (Christie et al., 2023; Kagoyire et al., 2023; May et al., 2023). Offspring of parents with PTE exposure were reported to display various mental health difficulties, including symptoms of PTSD, anxiety and the presence of self-damaging behaviors such as self-harm and substance abuse. Both parents and children shared the belief that these difficulties were partly related to maladaptive patterns of communication. In particular, accounts describe that communication was believed to negatively impact child well-being through the presence of negative emotions (i.e., anger, withdrawal) in day-to-day interactions, the transmission of negative worldviews and attitudes through conversation, and general patterns of silencing that stretch beyond the parental PTE. Communication was, however, never mentioned as the sole reason for the reported difficulties.

#### Trauma Transmission and Parent–Child Communication About PTEs

Twenty-three studies described details relevant to the relationship between parent–child communication about PTEs and intergenerational trauma transmission. Among quantitative records (*n* = 12), evidence of transmission was most commonly ascertained through one or multiple measures of child mental health, such as broad categories of psychiatric symptomatology (*n* = 4) or general well-being (*n* = 1), or more specific measures of PTSD (*n* = 3), anxiety (*n* = 2), depression (*n* = 3), distress (*n* = 2), guilt (*n* = 2), and attachment security (*n* = 2). Some studies also assessed transmission outside the direct scope of mental health through measures of PTE salience (*n* = 2), empathic responses (*n* = 1), and communication (*n* = 1).

In several studies, talking about PTEs with children was found to positively impact child well-being. When parents talked more frequently about PTEs, children displayed lower degrees of behavioral problems (Fargas-Malet & Dillenburger, 2016). Relatedly, Okner and Flaherty (1989), who assessed American and Israeli Holocaust survivor offspring, found that more discussions about parental PTEs were associated with lower reports of anxiety, depression, and demoralization among Americans but also more guilt, although Israeli offspring only faced lower demoralization. This implies that culture may play a role in how PTE communication impacts child well-being.

In line with the finding that talking about PTEs generally benefited the child, silencing and a lack of talking about PTEs were instead related to negative outcomes. A child’s apprehension to disclose their own PTEs could also influence child well-being. Kellermann (2008) found that a population of holocaust survivor offspring who had sought out therapy were characterized by a lack of talking about parental PTEs. Palgi et al. (2015) found that holocaust survivor offspring who were less willing to share their personal worries reported greater holocaust salience in their daily lives. In addition, children of parents with cancer who reported a higher specificity of discussed topics and a lack of emotion sharing encountered more PTSD symptoms (Huizinga et al., 2005).

The content and style of PTE communication also played a role in the differential impact on child well-being. Positive outcomes were found when PTE communication placed greater emphasis on factual knowledge, resulting in fewer PTSD symptoms, less distress, and greater empathic responses (Alkalay et al., 2020; Dalgaard et al., 2019; Wiseman et al., 2002). Meanwhile, a negative impact occurred when PTE-related communication lacked verbal communication and/or placed undue blame on the child for the parent’s experiences, resulting in more PTSD symptoms among offspring (Bonumwezi et al., 2024). One study that focused on comparing different PTE communication styles among refugees found that “unfiltered speech”—instances of children overhearing conversations about parental PTEs without having had any direct parent–child PTE communication—was associated with insecure attachment styles (Dalgaard et al., 2016).

Twelve studies obtained qualitative findings that provided additional insights into the relationship between parent–child PTE communication and trauma transmission. Several records showcased how PTE-exposed parents and their offspring believe in, acknowledge, and are conscious of the potential risk of trauma transmission. For many parents, the danger of potentially harming their children through their behavior was of great concern (Christie et al., 2023; Kagoyire et al., 2023; May et al., 2023; Sherman et al., 2015). Both parents and children asserted communication as one of several pathways believed to contribute to intergenerational trauma (Kagoyire et al., 2023). Aligning with previously mentioned quantitative findings, the approach, style, and content of PTE communication were believed to differentially impact child outcomes. Important factors believed to stimulate a positive influence on child well-being included the developmental appropriateness of utilized strategies (Wolf, 2019), mutual readiness to engage in communication about a parent’s traumatic past (Lin & Suyemoto, 2016), absence of incomplete, disjointed or fabricated narratives (Kagoyire et al., 2023), avoidance of over-disclosure, (Krauskopf et al., 2023; Prince, 1985), and congruence between information coming directly from the parent and other sources of information (i.e., family members, public sphere; Sinalo et al., 2021).

Noteworthy among qualitative records is the finding that the way parents communicated about their PTE could also shape how children spoke about and responded to their own struggles. Within households where parental PTEs were commonly silenced or subject to taboo, children appeared compliant to parental apprehension, avoiding relevant topics, and withholding their own worries to prevent causing distress (Krauskopf et al., 2023; May et al., 2023; Lin & Suyemoto, 2016; Sherman et al., 2015; Wolf, 2019). This transference of silence surrounding personal struggles could also present itself outside of the parent–child relationship. This was evidenced by Mcgaw et al. (2022), who found that Australian children of veteran parents with PTSD were reluctant to seek help and rarely turned to others when times were stressful. Altogether, these findings suggest that parental silence surrounding their PTEs may, in turn, cause children to adopt similar strategies surrounding their own difficulties encountered, including those that could be potentially traumatic.

## Discussion

The first aim of the current study was to explore general parent–child communication among parents who had experienced a PTE. It was found that parental PTE exposure can negatively affect both the quality and quantity of general parent–child communication, particularly due to the presence of parental anger and irritability, mutual withdrawal, and a lack of room for children to express personal worries. These findings map into previous studies that imply trauma exposure as a detriment to family (Dorrington et al., 2019) and social functioning (Scoglio et al., 2022). Although a majority of included studies asserted a negative impact, a few records did not find a relationship between PTE exposure and communication at all, implying that parent–child communication can also remain unaffected after a parent is exposed to a PTE. Interestingly, (life-threatening) illness acted as the PTE of interest (cancer; Gazendam et al., 2009; Huizinga et al., 2005) or played a significant role in the aftermath of the PTE endured (Chernobyl disaster; Bromet et al., 2000) among three of these four records. These findings may in part be explained by the nature of the PTE. Exposure to serious illness is often accompanied by a prospect of loss, which can intensify personal contact and emphasize the shared remembrance of positive memories (Caughlin et al., 2011; Gawinski et al., 2021). Other studies assessing family perspectives on communication have similarly found that perceptions of family communication can improve, though obstacles may still be encountered, particularly surrounding distressing emotions and uncertainty about the future (Fisher et al., 2017). Conclusively, general parent–child communication is often negatively impacted by parental PTE exposure, but whether and how this impact manifests may be partly dependent on the nature of the PTE.

Aside from investigating communication in general, the present review also explored how parents talk about their PTEs with their children. Results indicated that parents were overall more likely to speak up about their PTEs than remain completely silent, although silence was still common, and parents rarely disclosed fully with all details. Parental approaches to PTE communication were often motivated by a desire to protect children from harm that may stem from (not) disclosing parental PTEs and subsequent mental health consequences. Upon closer inspection, two noteworthy patterns emerge regarding differences in PTE communication between populations. First, Holocaust survivors and parents from non-Western populations did not display a disproportional amount of silencing or avoidance, contrary to previous findings that indicate both populations as prone to silence (Dalgaard et al., 2015; Wilson, 1985). Second, parents with PTSD, especially veteran parents, appeared to more commonly display strategies of silencing and avoidance surrounding their PTEs. This finding is not surprising given the nature of PTSD symptomatology: People with PTSD often exert great effort to avoid reminders of the trauma and risk facing substantial distress when made to revisit it (American Psychiatric Association, DSM-5 Task Force, 2013), rendering any instances of trauma disclosure as highly difficult and subject to avoidance. In addition, PTSD can give rise to negative thoughts and beliefs that, among parents, could also affect perceptions of their own child (i.e., belief that child is fragile and must be protected) and their own functioning as a parent (i.e., belief that their presence is harming the child; Christie et al., 2023; May et al., 2023). These beliefs may, in turn, cause PTE communication to be seen as risky or outright dangerous, resulting in silencing and avoidance. Altogether, these difficulties can shape a family environment that lacks engagement and communication, further limiting the room for PTE communication among families of parents with PTSD (Creech & Misca, 2017).

The final aim of the review focused on whether and how parent–child communication may contribute to intergenerational traumatization. In line with prior findings regarding the transmission of trauma across generations (Békés & Starrs, 2024; Kizilhan et al., 2021), both general communication and PTE communication were implied to act as pathways of transmission. In the case of general communication, a lower quality and quantity were unanimously deemed detrimental to child well-being, with the presence of negative emotions in daily interactions, the transmission of worldviews and attitudes, and patterns of silence/withdrawal being quoted as contributors to intergenerational traumatization. The impact of general parent–child communication on well-being was, however, studied by a comparatively small number of studies, highlighting the need for additional research to develop a comprehensive understanding.

Findings regarding PTE communication suggested that talking about PTEs was generally more beneficial to the child compared to little or no talking. However, classifying PTE communication merely according to quantity would be a gross oversimplification (Wolf, 2019), as the style and content of PTE communication play a valuable role in shaping outcomes. Positive outcomes were generally attributed to the parental approaches being developmentally sensitive and suitable to the child, for example, by emphasizing factual, age-appropriate communication, and avoiding gruesome content. Conversely, negative outcomes stemmed from conversations or patterns of communication that were subject to over-disclosure or placing blame on the child. Various studies also stressed the danger of children exclusively learning about the parental PTE through indirect means. In these instances, direct parent–child communication about the parental PTE was either discouraged or absent, but children would still obtain information by overhearing conversations (Dalgaard et al., 2016; Sherman et al., 2015; Rosenheck, 1986) or engaging with alternative sources (McGaw et al., 2022; Sinalo et al., 2021; Wolf, 2019). It is possible that obtaining knowledge in the absence of direct communication harms child well-being by resulting in fragmented, potentially distressing accounts of the parental PTE, which can be ill-suited or frustrating for the child (Dalgaard et al., 2019; DeNutte et al., 2022; Prince, 1985). Additionally, these instances may manifest or maintain a taboo surrounding the disclosure of adversity, interfering with a child’s personal coping methods and making them less likely to seek help (Krauskopf et al., 2023; Lin & Suyemoto, 2016; May et al., 2023). In sum, these findings underline that the contribution of parent–child communication to trauma transmission is best understood according to the content and context of these conversations. For an overview of key findings, see Table 2.

### Limitations

When interpreting the abovementioned findings, it is important to keep in mind several limitations that the present review was subject. First, the included records provided limited diversity in types of PTE exposure. A sizeable majority of studies (74%) regarded populations exposed to war or violent national conflicts, creating a relatively homogenous representation of PTE types in which commonly occurring PTEs such as sexual abuse, stalking, and the sudden loss of a loved one (Kessler et al., 2017) remained underrepresented. It is reasonable to acknowledge the potential that some PTE types may affect parent–child communication differentially, and that the current review therefore predominantly displays the effect of war-related PTEs, in particular (repeated) exposure to genocide, violent conflict, and military deployment. While some PTE types thus lack representation in this review, it was able to assess the impact of war-related PTEs in relation to a variety of contexts and events, providing a comprehensive representation of this PTE type.

Second, the bias assessment identified that a portion of the included studies were at risk of bias, which could impact the overall certainty of the evidence presented in the review (Hong et al., 2018). Some studies failed to adhere to all quality standards, most commonly due to concerns about methodological quality (specifically among mixed methods studies), a risk of nonresponse bias, and/or insufficiently reported information needed to conduct the bias assessment. Despite these shortcomings, a majority of studies fulfilled all quality criteria, allowing present findings to be grounded in a large body of reliable, qualitatively strong data. Additional bias may be caused by the source of data within studies. Although the final selection of studies provided ample representation of both parent and child perspectives on parent–child communication and intergenerational traumatization, a majority only collected data from either child or parent, thus enabling a risk of creating a one-sided view on the topics of interest within these dyads. Altogether, these factors could cause some studies to be biased in their findings, which should be taken into account during interpretation.

Finally, it should be noted that the included records did not represent children of all ages, warranting caution in generalizing present findings. The current review set out to accurately map parent–child communication and its relation to trauma transmission, carefully constructing inclusion and exclusion criteria to avoid potential confounding/overlap with related topics such as attachment and parenting styles. One of such criteria reasoned the exclusion of literature that only assessed nonverbal communication, inadvertently removing all studies of parent–child communication among newborns and infants. Early childhood has been implied to be the critical developmental phase for the transmission of trauma (Dozio et al., 2020) and could have provided valuable insights into this process. While this exclusion may have limited the generalizability, the review also finds strength in its representation of all other age groups, including adult children, a population that lacks visibility in research on parent–child (PTE) communication.

### Implications

Limitations notwithstanding, the present review carries valuable implications for scientific research and clinical practice (see Table 3). Arguably most central is the assertion that parents with PTE exposure are at risk for maladaptive parent–child (PTE) communication, which can in turn significantly contribute to the transmission of trauma by impacting child well-being and general functioning. Given the potentially hazardous influence maladaptive parent–child communication can exert on both child and parent (Grey et al., 2022), it would be highly beneficial to better embed general and PTE communication into the clinical treatment of traumatized parents and their offspring. To do so, clinicians and policymakers may consider the following considerations: Firstly, review results indicate that some PTE-exposed parents feel ill-equipped to approach difficult parent–child conversations due to not knowing how to engage and/or struggling to cope with strong (emotional) responses to the PTE (Christie et al., 2019; Sherman et al,, 2015). Education-based interventions such as psychoeducation could help inform parents about the impact of PTE exposure and mental health on parenting and guide them in developing healthier patterns of communication (Siegenthaler et al., 2012). When counseling or informing parents about parent–child PTE communication, it is important to take into account that there is no golden standard or one-size-fits-all strategy, and that outcomes are influenced by a variety of parent–related, child-related, and environmental factors. Findings suggest that certain patterns, particularly complete silencing, over-disclosure, and indirect disclosure (i.e., parents discussing PTEs with others while their children can overhear), are generally hazardous, while other strategies, particularly those emphasizing developmental appropriateness, are likely to yield more positive outcomes. Altogether, it would be beneficial to provide PTE-exposed parents with additional support surrounding parent–child (PTE) communication, placing emphasis on the importance of developmental appropriateness and formulating clinical counsel/interventions according to the specific wants, needs, and context of the parent and child.

This systematic review marks among the first attempts at synthesizing contemporary research about parent–child (PTE) communication between PTE-exposed parents and their (nonexposed) offspring, unearthing several novel insights that confirm and elucidate the complexity of these phenomena. Results speak in favor of previous calls to broaden conceptualization of parent–child PTE communication beyond categorical accounts of “telling” or “not telling” (Dalgaard et al., 2019; Wolf, 2019), instead working toward theoretical frameworks that perceive PTE communication as a dynamic, multifactorial process. Two findings would be valuable to take into account while paving the road toward such mapping. First, present findings identified several factors that can benefit or impair parent–child PTE communication, including belonging to “at-risk” populations, such as parents who are veterans, have PTSD, and/or were exposed to long-term, recurring PTE exposure (in early childhood). Second, parent–child PTE communication and its impact on child well-being may be influenced by the presence (or absence) of alternative sources of information. Multiple studies showcased how nonexposed parents, family members, peers, and community resources can be important sources of information about parental PTEs, influencing parental trajectories of disclosure and child well-being both positively and negatively (Houston et al., 2013; Sinalo et al., 2021). Weighing in the presence of such sources of information is therefore integral to comprehensively understanding how motivations, strategies, and outcomes of parent–child PTE communication take shape. Given the importance of “outside” information, policymakers and healthcare professionals could benefit from adopting trauma-informed models in communities. Such models could help ensure that community members, such as teachers, government workers, and local initiative leaders are adequately trained to recognize, address and educate about (parental) traumas (Avery et al., 2020; Champine et al., 2022), thereby potentially reducing the risk of maladaptive PTE communication between child and other sources of information while simultaneously providing (children of) PTE-exposed parents with beneficial skills to address PTEs. Altogether, these results sharpen current knowledge of parent–child (PTE) communication in the context of parental PTE exposure and enable innovation of theoretical models.

### Future Research

While research on communication between traumatized parents and their (nonexposed) offspring is steadily rising, there are still many opportunities to improve our current understanding. As addressed prior, most studies regarding parent–child (PTE) communication have been primarily focused on Western samples with repeated/long-term war- and conflict-related exposures. Currently, there remains a limited representation of other populations (i.e., refugees, first responders), PTE exposures (i.e., sexual violence, accidents, domestic abuse), and PTE frequencies (i.e., single case events), among which parent–child (PTE) communication may appear differently and would thus also be valuable to explore. In addition, these differential populations/exposures are often studied in isolation and rarely subjected to cross-comparison, creating barriers to synthesizing and generalizing findings, which remains a common problem among research on trauma and parenting in general (Meijer et al., 2023). Future research dedicated to investigating underrepresented populations/exposures would not only contribute to diversifying theoretical knowledge about parent–child communication among traumatized parents, but also enable meaningful comparisons within (i.e., similar PTE exposures across cultures) and between various samples (i.e., exposure to war in comparison to other exposures). These comparisons could, in turn, highlight important similarities and differences in parent–child (PTE) communication among various populations, further elucidating which factors and/or circumstances cause some parents to be “at risk” for parent–child communication-related challenges.

In order to facilitate the expansion and unification of research about parent–child communication within the context of parental PTEs, it would be beneficial to strive toward greater methodological rigor. To date, there are only a few instruments readily available for the sake of quantitatively measuring communication about parental PTEs, the majority of which are constructed with specific PTE exposures in mind (i.e., the Holocaust; Lichtman, 1984). The development of novel instruments for parent–child (PTE) communication would help address these shortcomings, bolstering the quality of assessments, making research on parent–child PTE communication more accessible, and enabling chances to more accurately compare different studies.

Finally, future studies should also consider further investigating the mechanisms that underlie intergenerational traumatization through means of (PTE) communication. Although contemporary literature seems to robustly imply that parent–child communication plays a role in the transmission of trauma, the exact pathways and factors that give rise to this detrimental outcome are studied only scarcely and often yield conflicting results. Beneficial directions for such additional research could include (further) investigating the impact of different PTE communication strategies on child well-being, the impact of parent–child (PTE) communication on children’s coping with personal concerns and PTEs, and the transmission of positive outcomes through (PTE) communication. Such research would be helpful in breaking the cycle of generational trauma transmission by identifying effective approaches for mitigating the long-term psychological impact on future generations. By empowering parents with tools to communicate trauma more constructively, these findings could foster resilience and emotional well-being, helping to interrupt patterns of intergenerational trauma and promoting healthier family dynamics.

### Conclusion

In conclusion, parental exposure to potentially traumatic events (PTEs) appears to have a clear negative impact on general parent–child communication. How parents speak about their PTEs in particular can vary greatly, with most common strategies resembling partial or modulated approaches to disclosure. Parent–child communication, both in general and about PTEs, could contribute to the transmission of trauma, stimulating negative but also positive outcomes on child well-being, depending on the content and context of communication. Findings carry meaningful implications for clinical practice and research dedicated to PTE-exposed parents and their offspring.

## Supplemental Material

sj-docx-1-tva-10.1177_15248380251343187 – Supplemental material for Parent–Child Communication after Parental Exposure to Potentially Traumatic Events: A Systematic Review Supplemental material, sj-docx-1-tva-10.1177_15248380251343187 for Parent–Child Communication after Parental Exposure to Potentially Traumatic Events: A Systematic Review by Dani de Beijer, Mèlanie Sloover, Karlijn Heesen and Elisa van Ee in Trauma, Violence, & Abuse

## References

[bibr1-15248380251343187] Afzal N. Ye S. Page A. C. Trickey D. Lyttle M. D. Hiller R. M. Halligan S. L. (2023). A systematic literature review of the relationship between parenting responses and child post-traumatic stress symptoms. European Journal of Psychotraumatology, 14(1), 2156053. 10.1080/20008066.2022.2156053 37052099 PMC9788707

[bibr2-15248380251343187] Alhassen S. Chen S. Alhassen L. Phan A. Khoudari M. De Silva A. Barhoosh H. Wang Z. Parrocha C. Shapiro E. Henrich C. Wang Z. Mutesa L. Baldi P. Abbott G. W. Alachkar A. (2021). Intergenerational trauma transmission is associated with brain metabotranscriptome remodeling and mitochondrial dysfunction. Communications Biology, 4(1), 783. 10.1038/s42003-021-02255-2 34168265 PMC8225861

[bibr3-15248380251343187] Alkalay S. Sagi-Schwartz A. Wiseman H. (2020). Increased empathy and helping behavior toward the mother in daughters of Holocaust survivors. Traumatology, 26(1), 84–95. 10.1037/trm0000211

[bibr4-15248380251343187] American Psychiatric Association, DSM-5 Task Force. (2013). Diagnostic and statistical manual of mental disorders: DSM-5™ (5th ed.). American Psychiatric Publishing, Inc. 10.1176/appi.books.9780890425596

[bibr5-15248380251343187] Avery J. C. Morris H. Galvin E. Misso M. Savaglio M. Skouteris H. (2020). Systematic review of school-wide trauma-informed approaches. Journal of Child & Adolescent Trauma, 14(3), 381–397. 10.1007/s40653-020-00321-1 34471456 PMC8357891

[bibr6-15248380251343187] Banneyer K. N. Koenig S. A. Wang L. A. Stark K. D. (2017). A review of the effects of parental PTSD: A focus on military children. Couple and Family Psychology: Research and Practice, 6(4), 274–286. 10.1037/cfp0000093

[bibr7-15248380251343187] Békés V. Starrs C. J. (2024). Assessing Intergenerational trauma transmission: development and psychometric properties of the Historical Intergenerational Trauma Transmission Questionnaire (HITT-Q). European Journal of Psychotraumatology, 15(1), 2329510. 10.1080/20008066.2024.2329510 38530844 PMC10967669

[bibr8-15248380251343187] Benjet C. Bromet E. Karam E. G. Kessler R. C. McLaughlin K. A. Ruscio A. M. Shahly V. Stein D. J. Petukhova M. Hill E. Alonso J. Atwoli L. Bunting B. Bruffaerts R. Caldas-de-Almeida J. M. de Girolamo G. Florescu S. Gureje O. Huang Y. Lepine J. P. , . . . Koenen K. C. (2016). The epidemiology of traumatic event exposure worldwide: results from the World Mental Health Survey Consortium. Psychological Medicine, 46(2), 327–343. 10.1017/S0033291715001981 26511595 PMC4869975

[bibr9-15248380251343187] Blankenship A. E. Drew A. L. Jacoby V. M. Zolinski S. K. Ojeda A. R. Dondanville K. A. Sharrieff A.-F. M. Yarvis J. Acker M. Blount T. H. McGeary C. A. Young-McCaughan S. Peterson A. L. Kritikos T. K. DeVoe E. R. (2024). Qualitative examination of homecoming experiences among active-duty military fathers during reintegration. Qualitative Social Work, 23(2), 298–313. 10.1177/14733250221150378

[bibr10-15248380251343187] Bonumwezi J. L. Grapin S. L. Uddin M. Coyle S. Habintwali D. Lowe S. R. (2024). Intergenerational trauma transmission through family psychosocial factors in adult children of Rwandan survivors of the 1994 genocide against the Tutsi. Social Science & Medicine, 348, 116837. 10.1016/j.socscimed.2024.116837 38579628

[bibr11-15248380251343187] Bromet E. J. Goldgaber D. Carlson G. Panina N. Golovakha E. Gluzman S. F. Gilbert T. Gluzman D. Lyubsky S. Schwartz J. E. (2000). Children’s well-being 11 years after the Chornobyl catastrophe. Archives of General Psychiatry, 57(6), 563–571. 10.1001/archpsyc.57.6.563 10839334

[bibr12-15248380251343187] Caughlin J. P. Mikucki-Enyart S. L. Middleton A. V. Stone A. M. Brown L. E. (2011). Being Open without Talking about It: A rhetorical/normative approach to understanding topic avoidance in families after a lung cancer diagnosis. Communication Monographs, 78(4), 409–436. 10.1080/03637751.2011.618141

[bibr13-15248380251343187] Champine R. B. Hoffman E. E. Matlin S. L. Strambler M. J. Tebes J. K. (2022). “What does it mean to be trauma-informed?”: A mixed-methods study of a trauma-informed community initiative. Journal of child and Family Studies, 31(2), 459–472. 10.1007/s10826-021-02195-9 35018088 PMC8736308

[bibr14-15248380251343187] Christie H. Hamilton-Giachritsis C. Alves-Costa F. Tomlinson M. Halligan S. L. (2019). The impact of parental posttraumatic stress disorder on parenting: a systematic review. European Journal of Psychotraumatology, 10(1), Article 1550345. 10.1080/20008198.2018.1550345 PMC6338266 30693071

[bibr15-15248380251343187] Christie H. Hamilton-Giachritsis C. McGuire R. Bisson J. I. Roberts N. P. Underwood J. F. G. Halligan S. L. (2023). Exploring the perceived impact of parental PTSD on parents and parenting behaviours—A qualitative study. Journal of Child and Family Studies, 32(11), 3378–3388. 10.1007/s10826-023-02614-z

[bibr16-15248380251343187] Cramm H. Godfrey C. M. Murphy S. McKeown S. Dekel R. (2022). Experiences of children growing up with a parent who has military-related post-traumatic stress disorder: A qualitative systematic review. JBI Evidence Synthesis, 20(7), 1638–1740. 10.11124/JBIES-20-00229 34710888

[bibr17-15248380251343187] Creech S. K. Misca G. (2017). Parenting with PTSD: A review of research on the influence of PTSD on parent-child functioning in military and veteran families. Frontiers in Psychology, 8, 1101. 10.3389/fpsyg.2017.01101 28713306 PMC5491843

[bibr18-15248380251343187] Dalgaard N. T. Diab S. Y. Montgomery E. Qouta S. R. Punamäki R.-L. (2019). Is silence about trauma harmful for children? Transgenerational communication in Palestinian families. Transcultural Psychiatry, 56(2), 398–427. 10.1177/1363461518824430 30702385

[bibr19-15248380251343187] Dalgaard N. T. Montgomery E. (2015). Disclosure and silencing: A systematic review of the literature on patterns of trauma communication in refugee families. Transcultural Psychiatry, 52(5), 579–593. 10.1177/1363461514568442 25656844 PMC4574085

[bibr20-15248380251343187] Dalgaard N. T. Todd B. K. Daniel S. I. Montgomery E. (2016). The transmission of trauma in refugee families: associations between intra-family trauma communication style, children’s attachment security and psychosocial adjustment. Attachment & Human Development, 18(1), 69–89. 10.1080/14616734.2015.1113305 26608277

[bibr21-15248380251343187] De Nutte L. De Haene L. Derluyn I . (2022). “They now know that they are children of war”: Forcibly abducted mothers and fathers balancing disclosure and silencing to their children born of war in Northern Uganda. Frontiers in Political Science, 4, 850969. 10.3389/fpos.2022.850969

[bibr22-15248380251343187] Dorrington S. Zavos H. Ball H. McGuffin P. Sumathipala A. Siribaddana S. Rijsdijk F. Hatch S. L. Hotopf M. (2019). Family functioning, trauma exposure and PTSD: A cross sectional study. Journal of Affective Disorders, 245, 645–652. 10.1016/j.jad.2018.11.056 30445390 PMC6362264

[bibr23-15248380251343187] Dozio E. Feldman M. Bizouerne C. Drain E. Laroche Joubert M. Mansouri M. Moro M. R. Ouss L. (2020). The transgenerational transmission of trauma: The effects of maternal PTSD in mother-infant interactions. Frontiers in Psychiatry, 11, 480690. 10.3389/fpsyt.2020.480690 33329072 PMC7733963

[bibr24-15248380251343187] Fargas-Malet M. Dillenburger K. (2016). Intergenerational transmission of conflict-related trauma in Northern Ireland: A behavior analytic approach. Journal of Aggression, Maltreatment & Trauma, 25(4), 436–454. 10.1080/10926771.2015.1107172

[bibr25-15248380251343187] Field N. P. Muong S. Sochanvimean V. (2013). Parental styles in the intergenerational transmission of trauma stemming from the Khmer Rouge regime in Cambodia. The American Journal of Orthopsychiatry, 83(4), 483–494. 10.1111/ajop.12057 24164520

[bibr26-15248380251343187] Fisher C. L. Wolf B. M. Fowler C. Canzona M. R. (2017). Experiences of “openness” between mothers and daughters during breast cancer: implications for coping and healthy outcomes. Psycho-oncology, 26(11), 1872–1880. 10.1002/pon.4253 27530810

[bibr27-15248380251343187] Fredman S. J. Beck J. G. Shnaider P. Le Y. Pukay-Martin N. D. Pentel K. Z. Monson C. M. Simon N. M. Marques L. (2017). Longitudinal associations between PTSD symptoms and dyadic conflict communication following a severe motor vehicle accident. Behavior Therapy, 48(2), 235–246. 10.1016/j.beth.2016.05.001 28270333 PMC6029245

[bibr28-15248380251343187] Gavidia-payne S. Denny B. Davis K. Francis A. Jackson M. (2015). Parental resilience: A neglected construct in resilience research. Clinical Psychologist, 19(3), 111–121. 10.1111/cp.12053

[bibr29-15248380251343187] Gawinski L. Stiel S. Schneider N. Herbst F. A. (2021). Communication in dyads of adult children at the end of life with their parents and parents at the end of life with their adult children: Findings from a mixed-methods study. Psycho-oncology, 30(9), 1535–1543. 10.1002/pon.5728 33982826

[bibr30-15248380251343187] Gazendam-Donofrio S. Hoekstra H. van der Graaf W. van de Wiel H. Visser A. Huizinga G. Hoekstra-Weebers J. , (2009). Parent-child communication patterns during the first year after a parent’s cancer diagnosis: the effect on parents’ functioning. Cancer, 115(18), 4227–4237. 10.1002/cncr.24502 19626654

[bibr31-15248380251343187] Grey E. B. Atkinson L. Chater A. Gahagan A. Tran A. Gillison F. B. (2022). A systematic review of the evidence on the effect of parental communication about health and health behaviours on children’s health and wellbeing. Preventive Medicine, 159, 107043. 10.1016/j.ypmed.2022.107043 35405179

[bibr32-15248380251343187] Lishner Freud E. Berant E . (2023). Holocaust communication, attachment orientation and distress among descendants of female holocaust survivors. Family Process, 62(4), 1655–1670. 10.1111/famp.12848 36582137

[bibr33-15248380251343187] Hartzell G. Stenson A. F. van Rooij S. J. H. Kim Y. J. Vance L. A. Hinrichs R. Kaslow N. Bradley B. Jovanovic T. (2022). Intergenerational effects of maternal PTSD: Roles of parenting stress and child sex. Psychological Trauma: Theory, Research, Practice, and Policy, 14(7), 1089–1098. 10.1037/tra0000542 31916804 PMC7343607

[bibr34-15248380251343187] Hassija C. M. Turchik J. A. (2016). An examination of disclosure, mental health treatment use, and posttraumatic growth among college women who experienced sexual victimization. Journal of Loss and Trauma, 21(2), 124–136. 10.1080/15325024.2015.10119

[bibr35-15248380251343187] Hong Q. H. Pluye P. Fàbregues S. Bartlett S. Boardman F. Cargo M. Dagenais P. Gagnon M. Griffiths F. Nicolau B. O’Cathain A. Rousseau M. Vedel I. (2018). Mixed methods appraisal tool [User guide]. Canadian Intellectual Property Office. http://mixedmethodsappraisaltoolpublic.pbworks.com/w/file/fetch/127916259/MMAT_2018_criteriamanual_2018-08-01_ENG.pdf 10.1016/j.jclinepi.2019.03.008 30905698

[bibr36-15248380251343187] Houston J. B. Pfefferbaum B. Sherman M. D. Melson A. G. Brand M. W. (2013). Family communication across the military deployment experience: Child and spouse report of communication frequency and quality and associated emotions, behaviors, and reactions. Journal of Loss and Trauma, 18(2), 103–119. 10.1080/15325024.2012.684576

[bibr37-15248380251343187] Huizinga G. A. Visser A. van der Graaf W. T. Hoekstra H. J. Hoekstra-Weebers J. E. (2005). The quality of communication between parents and adolescent children in the case of parental cancer. Annals of Oncology, 16(12), 1956–1961. 10.1093/annonc/mdi395 16143592

[bibr38-15248380251343187] Jeyasundaram J. Cao L. Y. D. Trentham B. (2020). Experiences of intergenerational trauma in second-generation refugees: Healing through occupation. Canadian Journal of Occupational Therapy, 87(5), 412–422. 10.1177/0008417420968684 33256470

[bibr39-15248380251343187] Kagoyire M. G. Kangabe J. Ingabire M. C. (2023). A calf cannot fail to pick a colour from its mother: Intergenerational transmission of trauma and its effect on reconciliation among post-genocide Rwandan youth. BMC Psychology, 11(1), 104. 10.1186/s40359-023-01129-y 37029441 PMC10080878

[bibr40-15248380251343187] Kav-Venaki S. Nadler A. Gershoni H. (1983). Sharing past traumas: A comparison of communication behaviours in two groups of Holocaust survivors. International Journal of Social Psychiatry, 29(1), 49–59. 10.1177/002076408302900106 6840991

[bibr41-15248380251343187] Kellermann N. P. , (2001). Transmission of Holocaust trauma—An integrative view. Psychiatry: Interpersonal and Biological Processes, 64(3), 256–267. 10.1521/psyc.64.3.256.1846 11708051

[bibr42-15248380251343187] Kellermann N. P. (2008). Transmitted Holocaust trauma: curse or legacy? The aggravating and mitigating factors of Holocaust transmission. The Israel Journal of Psychiatry and Related Sciences, 45(4), 263–270. 19439826

[bibr43-15248380251343187] Kessler R. C. Aguilar-Gaxiola S. Alonso J. Benjet C. Bromet E. J. Cardoso G. Degenhardt L. de Girolamo G. Dinolova R. V. Ferry F. Florescu S. Gureje O. Haro J. M. Huang Y. Karam E. G. Kawakami N. Lee S. Lepine J. P. Levinson D. Navarro-Mateu F. , . . . Koenen K. C. (2017). Trauma and PTSD in the WHO World Mental Health Surveys. European Journal of Psychotraumatology, 8(Suppl. 5), 1353383. 10.1080/20008198.2017.1353383 29075426 PMC5632781

[bibr44-15248380251343187] Kevers R. de Smet S. Rober P. Rousseau C. De Haene L. (2024). Silencing or silent transmission? An exploratory study on trauma communication in Kurdish refugee families. Family process, 63(4), 2324–2346. 10.1111/famp.12996 38566251

[bibr45-15248380251343187] Kizilhan J. I. Noll-Hussong M. Wenzel T. (2021). Transgenerational transmission of trauma across three generations of Alevi Kurds. International Journal of Environmental Research and Public Health, 19(1), 81. 10.3390/ijerph19010081 35010342 PMC8751140

[bibr46-15248380251343187] Krauskopf I. E. Bates G. W. Cook R. (2023). Children of holocaust survivors: The experience of engaging with a traumatic family history. Genealogy, 7(1), 20. 10.3390/genealogy7010020

[bibr47-15248380251343187] Lewis-O'Connor A. Warren A. Lee J. V. Levy-Carrick N. Grossman S. Chadwick M. Stoklosa H. Rittenberg E . (2019). The state of the science on trauma inquiry. Women’s Health, 15, 1745506519861234. 10.1177/1745506519861234 PMC6713964 31456510

[bibr48-15248380251343187] Leys C. Fossion P. Verlarde S. A. Miller S. Kotsou I. , (2024). The effects of the intergenerational transmission of the Holocaust trauma on family functioning, resilience, anxiety and depression: A case-control study. European Journal of Trauma & Dissociation, 8(2), Article 100394. 10.1016/j.ejtd.2024.100394

[bibr49-15248380251343187] Lichtman H. (1984). Parental communication of Holocaust experiences and personality characteristics among second-generation survivors. Journal of Clinical Psychology, 40(4), 914–924. https://doi.org/10.1002/1097-4679(198407)40:4<914::aid-jclp2270400408>3.0.co;2-u 6480857 10.1002/1097-4679(198407)40:4<914::aid-jclp2270400408>3.0.co;2-u

[bibr50-15248380251343187] Lin N. J. Suyemoto K. L. (2016). So you, my children, can have a better life: A Cambodian American perspective on the phenomenology of intergenerational communication about trauma. Journal of Aggression, Maltreatment & Trauma, 25(4), 400–420. 10.1080/10926771.2015.1133748

[bibr51-15248380251343187] May K. Van Hooff M. Doherty M. Carter D. (2023). Experiences of parental PTSD for children aged 9–17 in military and emergency first responder families. Journal of Child and Family Studies, 32(12), 3816–3834. 10.1007/s10826-023-02669-y

[bibr52-15248380251343187] McGaw V. E. Reupert A. E. (2022). “Do not talk about that stuff”: Experiences of Australian youth living with a veteran parent with PTSD. Traumatology, 28(1), 24–30. 10.1037/trm0000317

[bibr53-15248380251343187] Meijer L. Franz M. R. Deković M. van Ee E. Finkenauer C. Kleber R. J. van de Putte E. M. Thomaes K. (2023). Towards a more comprehensive understanding of PTSD and parenting. Comprehensive Psychiatry, 127, 152423. 10.1016/j.comppsych.2023.152423 37722204

[bibr54-15248380251343187] O’Brien J. Creaner M. Nixon E. (2019). Experiences of fatherhood among men who were sexually abused in childhood. Child Abuse & Neglect, 98, 104177. 10.1016/j.chiabu.2019.104177 31655250

[bibr55-15248380251343187] Ochoa L. G. Fernandez A. Lee T. K. Estrada Y. Prado G. (2022). The intergenerational impact of adverse childhood experiences on hispanic families: The mediational roles of parental depression and parent-adolescent communication. Family Process, 61(1), 422–435. 10.1111/famp.12652 33880753 PMC9509697

[bibr56-15248380251343187] Okner D. F. Flaherty J. (1989). Parental communication and psychological distress in children of holocaust survivors: A comparison between the U.S. and Israel. International Journal of Social Psychiatry, 35(3), 265–273. 10.1177/002076408903500307 2583959

[bibr57-15248380251343187] Overstreet C. Berenz E. C. Kendler K. S. Dick D. M. Amstadter A. B. (2017). Predictors and mental health outcomes of potentially traumatic event exposure. Psychiatry Research, 247, 296–304. 10.1016/j.psychres.2016.10.047 27940325 PMC5921931

[bibr58-15248380251343187] Page M. J. McKenzie J. E. Bossuyt P. M. Boutron I. Hoffmann T. C. Mulrow C. D. Shamseer L. Tetzlaff J. M. Akl E. A. Brennan S. E. Chou R. Glanville J. Grimshaw J. M. Hróbjartsson A. Lalu M. M. Li T. Loder E. W. Mayo-Wilson E. McDonald S. McGuinness L. A. , . . . Moher D. (2021). The PRISMA 2020 statement: An updated guideline for reporting systematic reviews. BMJ, 372, n71. 10.1136/bmj.n71 PMC8005924 33782057

[bibr59-15248380251343187] Palgi Y. Shrira A. Ben-Ezra M. (2015). Family involvement and Holocaust salience among offspring and grandchildren of Holocaust survivors. Journal of Intergenerational Relationships, 13(1), 6–21. 10.1080/15350770.2015.992902

[bibr60-15248380251343187] Prince R. M. (1985). Second generation effects of historical trauma. Psychoanalytic Review, 72(1), 9–29. 3922007

[bibr61-15248380251343187] Ratcliffe M. (2022). Trauma, Language, and Trust. In Bortolan A. Magrì E. (Ed.), Empathy, intersubjectivity, and the social world: the continued relevance of phenomenology. Essays in honour of Dermot Moran (pp. 323–342). De Gruyter. 10.1515/9783110698787-017

[bibr62-15248380251343187] Rosenheck R. (1986). Impact of posttraumatic stress disorder of World War II on the next generation. The Journal of Nervous and Mental Disease, 174(6), 319–327. 10.1097/00005053-198606000-00001 3711873

[bibr63-15248380251343187] Saleh M. F. Rabaia Y. Balsam C. Amro Z. Kassis S. Giacaman R. (2019). Fathers detained, contact restrained: Experiences of Palestinian children visiting their fathers in Israeli detention. Child Abuse & Neglect, 96, 104071. 10.1016/j.chiabu.2019.104071 31400603

[bibr64-15248380251343187] Schlaudt V. A. Bosson R. Williams M. T. German B. Hooper L. M. Frazier V. Carrico R. Ramirez J. (2020). Traumatic experiences and mental health risk for refugees. International Journal of Environmental Research and Public Health, 17(6), 1943. 10.3390/ijerph17061943 32188119 PMC7143439

[bibr65-15248380251343187] Scoglio A. A. J. Reilly E. D. Girouard C. Quigley K. S. Carnes S. Kelly M. M. , (2022). Social Functioning in Individuals With Post-Traumatic Stress Disorder: A Systematic Review. Trauma, Violence & Abuse, 23(2), 356–371. 10.1177/1524838020946800 32812513

[bibr66-15248380251343187] Sherman M. D. Larsen J. Straits-Troster K. Erbes C. Tassey J. (2015). Veteran-child communication about parental PTSD: A mixed methods pilot study. Journal of Family Psychology, 29(4), 595–603. 10.1037/fam0000124 26374938

[bibr67-15248380251343187] Siegenthaler E. Munder T. Egger M. (2012). Effect of preventive interventions in mentally ill parents on the mental health of the offspring: systematic review and meta-analysis. Journal of the American Academy of Child and Adolescent Psychiatry, 51(1), 8–17. e8. 10.1016/j.jaac.2011.10.018 22176935

[bibr68-15248380251343187] Sinalo C. Irakoze P. C. Veale A. (2021). Disclosure of genocide experiences in Rwandan families: Private and public sources of information and child outcomes. Peace and Conflict: Journal of Peace Psychology, 27(4), 642–653. 10.1037/pac0000521

[bibr69-15248380251343187] Siqveland J. Hafstad G. S. Tedeschi R. G. (2012). Posttraumatic growth in parents after a natural disaster. Journal of Loss and Trauma, 17(6), 536–544. 10.1080/15325024.2012.678778

[bibr70-15248380251343187] Sloover M. Stoltz S. E. M. J. van Ee E . (2024). Parent-child communication about potentially traumatic events: A systematic review. Trauma, Violence & Abuse, 25(3), 2115–2127. 10.1177/15248380231207906 PMC11155229 37946404

[bibr71-15248380251343187] Spiel S. Szymanski K. Bornstein R. (2023). Intergenerational trauma, dependency, and detachment. The Journal of Nervous and Mental Disease, 211(9), 679–685. 10.1097/NMD.0000000000001682 37399584

[bibr72-15248380251343187] Tarabay J. Golm D. (2024). The transmission of intergenerational trauma and protective factors in survivors of the Lebanese civil war and their adult offspring. International Journal of Intercultural Relations, 99, Article 101592. 10.1016/j.ijintrel.2024.101952

[bibr73-15248380251343187] Vallières F. Hyland P. Murphy J. (2021). Navigating the who, where, what, when, how and why of trauma exposure and response. European Journal of Psychotraumatology, 12(1), 1855903. 10.1080/20008198.2020.1855903 34025911 PMC8128124

[bibr74-15248380251343187] van Ee E. Kleber R. J. Jongmans M. J. (2016). Relational patterns between caregivers with PTSD and their nonexposed children: A Review. Trauma, Violence, & Abuse, 17(2), 186–203. 10.1177/1524838015584355 25964276

[bibr75-15248380251343187] Wilson A. (1985). On silence and the holocaust: A contribution to clinical theory. Psychoanalytic Inquiry, 5(1), 63–84. 10.1080/07351698509533576

[bibr76-15248380251343187] Wiseman H. Barber J. P. Raz A. Yam I. Foltz C. Livne-Snir S. (2002). Parental communication of Holocaust experiences and interpersonal patterns in offspring of Holocaust survivors. International Journal of Behavioral Development, 26(4), 371–381. 10.1080/01650250143000346

[bibr77-15248380251343187] Wolf D. L. (2019). Postmemories of joy? Children of Holocaust survivors and alternative family memories. Memory Studies, 12(1), 74–87. 10.1177/1750698018811990

[bibr78-15248380251343187] Zhou A. Ryan J. (2023). Biological embedding of early-life adversity and a scoping review of the evidence for intergenerational epigenetic transmission of stress and trauma in humans. Genes, 14(8), 1639. 10.3390/genes14081639 37628690 PMC10454883

