# Supplementary material for: Parent–Child Communication after Parental Exposure to Potentially Traumatic Events: A Systematic Review
Source: Trauma Violence Abuse. 2025 Jun 27;27(4):1251–69. doi: 10.1177/15248380251343187 (PMC13578672; doi:10.1177/15248380251343187)
Supplement: Supplementary file 1 [file sj-docx-1-tva-10.1177_15248380251343187.docx]

**Supplemental Material File 1.** Database Search String.

(“PTSD” OR “posttraumatic stress disorder” OR psychotrauma OR post-trauma OR trauma OR traumatic events OR injur*”) AND (“parent” OR “famil*” OR “mother” OR “maternal” OR “father” OR “paternal” OR “Intergenerational” OR “intergenerational”) AND (“child*” OR “kid” OR “adolescen*”) AND (“communicat*” OR “disclos*” OR “talk*” OR “convers*” OR “express*”).
